# Physio-avatar EB: aftereffects in error learning with EMG manipulation of first-person avatar experience

**DOI:** 10.3389/fbioe.2024.1421765

**Published:** 2024-10-09

**Authors:** Tetsuya Ando, Kazuhiro Matsui, Yuto Okamoto, Keita Atsuumi, Kazuhiro Taniguchi, Hiroaki Hirai, Atsushi Nishikawa

**Affiliations:** ^1^ Graduate School of Engineering Science, Osaka University, Toyonaka, Japan; ^2^ Graduate School of Information Sciences, Hiroshima City University, Hiroshima, Japan; ^3^ Faculty of Human Ecology, Yasuda Women’s University, Hiroshima, Japan

**Keywords:** rehabilitation, virtual reality, error learning, internal model, electromyogram, biofeedback

## Abstract

**Introduction:**

Many studies have investigated the manipulation of a virtual upper arm using electromyogram (EMG); however, these studies primarily used a machine learning model or trigger control for this purpose. Furthermore, most of them could only display the constant motion of the virtual arm because the motion to be displayed was selected by pattern recognition or trigger control. In addition, these studies did not examine changes in the electromyographic signals after experiencing the virtual arm. By contrast, we propose a real-time, continuous, learning-free avatar that manipulates the virtual arm with electromyogram signals or physio-avatar EMG biofeedback (EB). The goal of the physio-avatar EB system is to induce physiological changes through experiential interactions.

**Methods:**

We explored the possibility of changing motor control strategies by applying the system to healthy individuals as a case study. An intervention method that provided an experience of a body different from one’s own was conducted on seven participants using a time-invariant calculation algorithm to determine the joint angles of the avatar. Control strategies for an indicator of the equilibrium point in the baseline and adaptation phases were determined to evaluate the physio-avatar EB intervention effect. The similarity of these BL and adaptation control strategies compared to those used during the washout period was assessed using the coefficient of determination. The accuracy and reliability of the virtual reality (VR) system were evaluated by comparison with existing studies and the required specs.

**Results and Discussion:**

Changes in motor control strategies due to the physio-avatar EB system were observed in four experiments, where the participants gradually returned to their pre-intervention control strategies. This result can be attributed to the aftereffects caused by error learning. This implies that the developed system influenced their motor control strategies. The number of EMG acquisition bits was 16 bits, and the sampling rate was 1,000 Hz. The refresh rate of the head-mounted display was 90 Hz, and its resolution was 1440×1600 for a single eye. Additionally, the simulation frame rate was 30 FPS. These values were adequate compared to existing studies and required specs. The essential contribution of this study is the development of an avatar that is controlled by a different method than has been used in previous studies and the demonstration of changes in a subject’s muscle activity after they experience an avatar. In the future, the clinical efficacy of the proposed system will be evaluated with actual patients.

## 1 Introduction

The advancement of virtual reality (VR) has made it easier for general consumers to operate avatars representing themselves in VR spaces, leading to an increase in avatar-based research. With the diversification of technologies using avatars, the physical and cognitive modifications induced by avatar experiences need to be investigated (Hagita, 2022). Previous studies have suggested the possibility of cognitive modifications resulting from avatar experiences. Keizer et al. (2016) aimed to resolve the discrepancy between the actual body and the self-body image, which causes eating disorders, by inducing a sense of body ownership toward avatars with different body types. Ratan and Sah (2015) experimentally investigated the stereotype threat effect after gaming based on the avatar gender and found an improvement in math scores among women using male avatars. These studies indicate that the impact of avatar experiences can be retained in real spaces, potentially affecting brain processing. Avatar use can induce changes in the brain concerning bodily movement control and can thus be applied to stroke rehabilitation. This is supported by reports stating that avatar experiences induce a sense of body ownership and agency (Preston and Ehrsson, 2016; Waltemate et al., 2018; Tieri et al., 2015), suggesting the possibility of mistaking an avatar for one’s own body. An individual may undergo “error learning” or “reinforcement learning” by experiencing their avatar.

In the error learning process, one’s internal model, believed to be in the cerebellum, is modified to reduce discrepancies between the executed (or outcome) motion trajectory and the expected motion trajectory. During the learning process, stiffness is high initially; virtual trajectory control is prioritized, but model-like control is subsequently implemented, thus reducing performance error and enabling feedforward motion control (Osu et al., 2002). The aftereffects of error learning are those previously induced for rehabilitation through error augmentation therapy using robots or VR (Lo et al., 2010; Liu et al., 2018; Wei et al., 2005; Abdollahi et al., 2013; Porta et al., 2021). Avatar experiences can be used for rehabilitation if the aftereffects of error learning occur. Furthermore, amplifying weak electromyogram (EMG) signals can enable the use of reinforcement learning in systems applied to patients with paralysis. Reinforcement learning focuses on the appropriateness of the executed motion outcome rather than errors during motion; successful task completion is rewarded, such as with a sense of achievement. Important factors in reinforcement learning include the difference between actual and predicted rewards, task difficulty, and exploration opportunities. Learning advances when the obtained reward exceeds the predicted reward, so task difficulty should be set appropriately, and movement methods should be explored to maximize rewards (Schultz et al., 1993; Schultz et al., 1997; Tanaka et al., 2016; Dobkin et al., 2010). Thus, amplifying weak EMG signals to enable an individual to experience movement through an avatar as a substitute for paralyzed limbs can lead to reinforcement learning. Many studies have used EMG signals in virtual reality (Tigrini et al., 2024; Tigrini et al., 2023); KiNvis™ is a reinforcement learning-based therapeutic method that utilizes EMG signals as triggers for avatar motion. The avatar moves when the EMG signals obtained from the muscles performing the set movement exceed a set threshold (Kaneko, 2016).

We propose an EMG biofeedback (EB) system that projects an arm-shaped avatar in the VR space within a head-mounted display (HMD), allowing for the continuous, real-time EMG-based control of the avatar. This system is modeled based on the findings of our previous studies, where we obtained a human neuromuscular skeletal model by expressing stimulation intensities to opposing muscles as two variables: the electrical agonist–antagonist (EAA) muscles sum and ratio (Nagai et al., 2019; Matsui et al., 2014; Matsui et al., 2015; Matsui et al., 2022; Suzuki et al., 2024). Error learning can be implemented using this system by setting the avatar’s characteristics to differ from the user’s own, whereas reinforcement learning can be executed by amplifying the EMG signals for patients with weak signals. Several studies demonstrated the acquisition of EMG signals and controlling of virtual hand models supporting hand motion rehabilitation. Gieser et al. (2017), Yang et al. (2017), and MINDROVE (2024) used an armband device that acquires the EMG signals of eight muscles, estimates hand movements using a machine learning approach, and displays constant movements, like the KiNvis™, according to estimated results. The number of bits corresponding to the accuracy of the EMG data measurement was unknown, and the EMG acquisition rates ranged from 200 to 500 Hz. Because Gieser said that the EMG activity rate could be as high as 450 Hz (Gieser et al., 2017), this sampling rate was insufficient based on the sampling theorem. Suryanarayanan et al. acquired EMG signals for a single biceps muscle and estimated joint angles using a learning method (Suryanarayanan and Reddy, 1997) and displayed the angles of the virtual arm by EMG changes. EMG signals were acquired with 12 bits, and the sampling rate was 2000 Hz. This sampling rate is somewhat high. These reported devices do not have a fixed display system, and their refresh rate depends on the used display unit. In contrast to these studies, our physio-avatar EB is unique, in that it uses a real-time, continuous, learning-free model to manipulate the virtual arm using EMG with a sampling rate of 1,000 Hz.

This study aims to validate the system’s effectiveness as a precursor to future applications for patients with post-stroke paralysis. We conducted an experiment on seven healthy individuals to examine the impact of avatar experiences on motor control strategies before and after the intervention as a case study. Although reinforcement learning cannot be confirmed without testing with patients with paralysis, we first used this system to verify whether healthy individuals can adapt to the movement characteristics of the model-defined avatar and learn the characteristics associated with the defined model in the internal model. The system aims to enable both error learning and reinforcement learning using avatars. In addition, we evaluate the developed physio-avatar EB by comparing it with existing medical devices. We used the number of EMG acquisition bits and sampling rate for accuracy and reliability and used the HMD refresh rate and resolution for realistic quality.

### 1.1 Physio-avatar EB

Avatar therapy is the use of avatars for rehabilitation. A physio-avatar, as we have named it, is an avatar designed to induce physiological changes in humans through experience. In this study, we use EB with a physio-avatar, so the studied avatar is called a physio-avatar EB.

### 1.2 System

**FIGURE 1:**
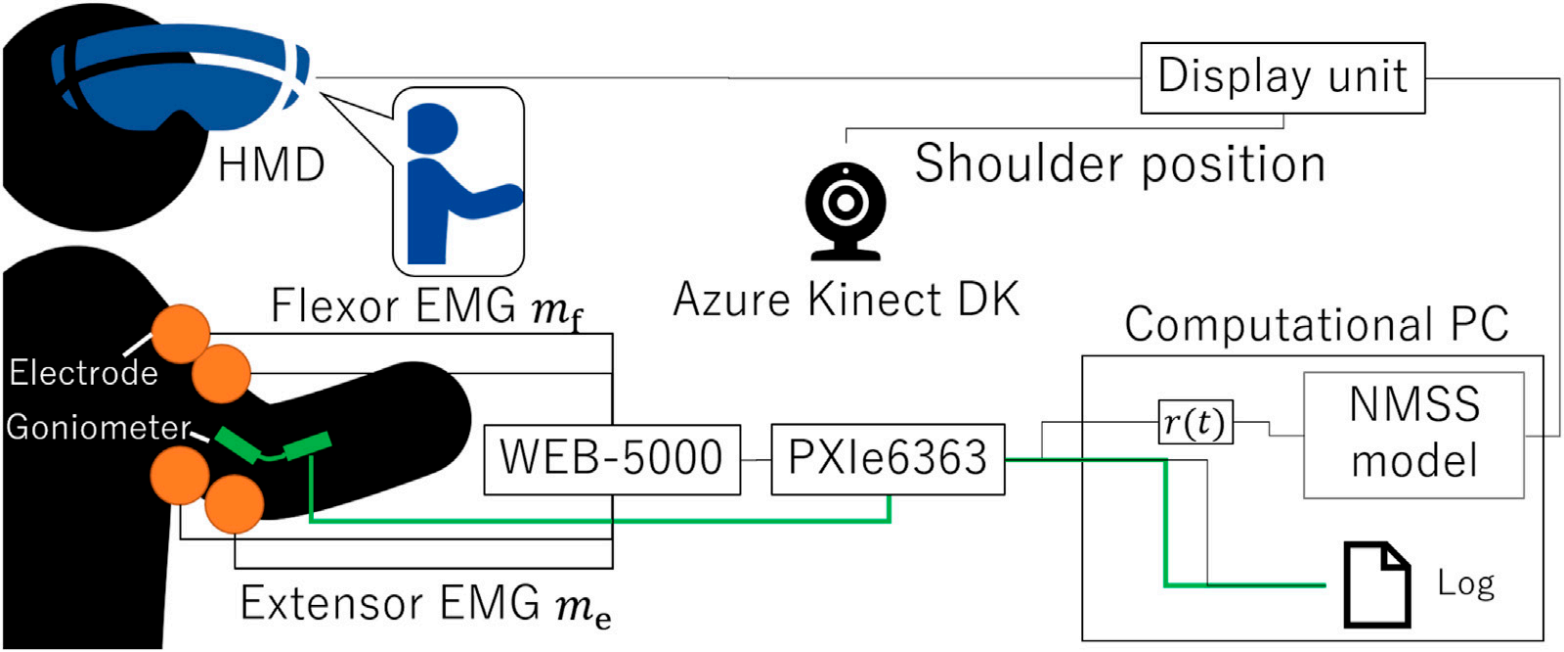
System block diagram illustrating the system design concept.

We developed the physio-avatar EB system (Figure 1), which enables users to experience a first-person avatar for one-degree-of-freedom (1-DOF) elbow joint movements (Okamoto et al., 2024). This system is primarily divided into a computational unit and a display unit. The computational unit is constructed using LabVIEW (NI) (NATIONAL INSTRUMENTS CORP., 2024a) on a computational PC and a data acquisition board PXIe6363 (NI) for 16-bit analog–digital conversion (NATIONAL INSTRUMENTS CORP., 2024b). Specifically, analog signals from an EMG acquisition device (WEB-5000, Nihon Kohden) (Masumoto et al., 2004) and an angle acquisition sensor goniometer (SG150, Biometrics Ltd.) (Biometrics Ltd, 2024) are converted into digital signals. These signals are then processed and computed in LabVIEW. The display unit consists of Unity (Unity Technologies) (Unity Technologies, 2024) installed on a display PC, Azure Kinect DK (Microsoft) (Microsoft, 2024), and a VIVE Pro (HTC) (HTC Corporation, 2024) HMD. The goal is for a user to achieve a sense of body ownership toward the avatar. The Azure Kinect DK kit is used to align the avatar in the VR space in the HMD according to the participant’s shoulder joint position and angle. The angle, dependent on the avatar characteristics calculated by the PC based on the EMG input, is sent to the display unit *via* transmission control protocol/internet protocol communication. In this study, the biceps brachii muscle was used as the flexor muscle and the triceps brachii muscle as the extensor muscle, and the positions of the surface electrodes were fixed based on the literature (Doheny et al., 2008). The ground electrode was attached to the head of the elbow.

Based on our previous research (Nagai et al., 2019; Matsui et al., 2014; Matsui et al., 2015; Matsui et al., 2022; Suzuki et al., 2024), the agonist–antagonist muscle ratio (AA ratio), an indicator of the equilibrium point in the human motion control, is input to the neuromuscular system (NMS) + musculoskeletal system (MSS) model (NMSS model) to calculate the virtual angle. The NMSS model integrates the transfer function of the NMS $G_{\text{NM}}\left(s\right)$ with the transfer function of the MSS $G_{\text{MS}}\left(s\right)$ to create a two-stage, time-invariant infinite impulse response filter + gain + dead time. NMSS model parameters change with joint stiffness (Matsui et al., 2014; Matsui et al., 2022; Gong et al., 2020), necessitating the alteration of filter coefficients based on the agonist–antagonist muscle sum (AA sum), an indicator of joint stiffness. However, this study uses a time-invariant filter. The transfer functions of the NMS and MSS are shown in Equations 1, 2, respectively.

$$G_{\text{NM}}\left(s\right)=K_{\text{NM}}\frac{\omega_{\text{nNM}}^{2}}{s^{2}+2\zeta_{\text{NM}}\omega_{\text{nNM}}s+\omega_{\text{nNM}}}e^{-L_{\text{NM}}s}.$$ (1)

$$G_{\text{MS}}\left(s\right)=K_{\text{MS}}\frac{\omega_{\text{nMS}}^{2}}{s^{2}+2\zeta_{\text{MS}}\omega_{\text{nMS}}s+\omega_{\text{nMS}}^{2}}.$$ (2)

Here, $G_{\text{NM}}\left(s\right)$ , $K_{\text{NM}}$ , $\omega_{\text{nNM}}$ , $\zeta_{\text{NM}}$ , and $L_{\text{NM}}$ represent the transfer function, gain, natural frequency, damping coefficient, and dead time of the NMS, respectively. $G_{\text{MS}}\left(s\right)$ , $K_{\text{MS}}$ , $\omega_{\text{nMS}}$ , and $\zeta_{\text{MS}}$ represent the transfer function, gain, natural frequency, and damping coefficient of the MSS, respectively, and serve as filter coefficients. The background for these transfer functions is our previous study, which examined whether the nonlinearities of the neuromusculoskeletal system could be approximated using a simple linear model and electrical stimulations. Matsui et al. (2014) showed that the neural and muscular system, which is an active system with nonlinear muscles and nerves, could be approximated using a simple linear model that utilizes the EAA ratio and EAA sum as the electrical stimulation parameters. These parameters correspond to the electromyographic parameters AA ratio and AA sum used in the present study. This neural and muscular system represents the NMS in the present study. Similarly, Matsui et al. (2015) showed that a passive system comprising nonlinear muscles and a skeleton, such as the muscular and skeletal system, could be approximated using a simple linear model that utilizes the EAA ratio, the EAA sum, and external forces applied to muscles and a skeleton by the robot arm. This muscular and skeletal system represents the MSS in the present study. The physio-avatar EB is an application that uses a model to linearly approximate the nonlinearity of the neuromusculoskeletal system based on previous studies. The maximum allowable values for the natural frequency and damping constant in each filter are 70.0 and 2.0, respectively.

In this system, due to the time needed to calculate the virtual angle from the AA ratio input (approximately 3 [ms] ), the EMG sampling rate is set to 1,000 [Hz] , and the virtual angle is computed at 100 [Hz] . The EMG signals from the extensors and flexors are processed using a 10 [Hz] high-pass filter and a 100 [Hz] low-pass filter, followed by rectification and processing using a 22 [Hz] low-pass filter. These values are then converted into %40 NVC (40 [N] voluntary contraction), normalized for the EMG during 40 [N] force generation. The concepts of the AA ratio and AA sum have been studied for a long time. As measurement variables, they satisfactorily explain the coordination of the agonist and antagonist muscles (Hirai et al., 2015). In these studies, the EMG measure was calibrated from the maximum force generated by a participant to calculate the sum and ratio of the agonist and antagonist muscle activities. In the present study, given the nonlinearity of muscles in high-intensity contractions (Lawrence and De Luca, 1983) and considering that the task to be performed requires only a small amount of force, the EMG is calibrated with a force of 40 N, which is less than the maximum exerted force so that the sum and ratio of the agonist and antagonist muscle activities can be calculated.

%40 NVC normalizes the EMG of flexors and extensors during activity to the EMG at 40 [N] exertion. For experimental preparation, the EMG signals from flexors at 40 [N] exertion in the flexion direction and the EMG signals from extensors at 40 [N] exertion in the extension direction are obtained and used for normalization. The average EMG signals during 40 ± 3 [N] exertion at a fixed joint angle between 90 [deg] and 120 [deg] in the extension direction for 5 s serve as the EMG at 40 [N] exertion. The AA sum s(t) and AA ratio r(t) are calculated using %40 NVC for extensors $m_{\text{e}}\left(t\right)$ and flexors $m_{\text{f}}\left(t\right)$ (Equations 3, 4).

$$s\left(t\right)=m_{\text{e}}\left(t\right)+m_{\text{f}}\left(t\right).$$ (3)

$$r\left(t\right)=\frac{m_{\text{e}}\left(t\right)}{s\left(t\right)}.$$ (4)

The calculated AA ratio and its difference from the initial AA ratio, $r^{\prime }\left(t\right)$ , are input to the NMSS model at a sampling rate of 100 Hz, resulting in the output value Δθ(t) . Because Δθ(t) represents the change in the AA ratio from its initial state, the virtual angle θ(t) is calculated by adding the joint angle at system start-up. This angle is then applied to the avatar in the display unit.

For the accuracy and reliability of physio-avatar EB, the number of EMG acquisition bits was 16, and the sampling rate was 1,000 Hz. This number of acquisition bits for an EMG was considered sufficient because 16-bit resolution is used in commercial medical devices, for example, the Tringo series (Delsys Incorporated) (Gieser et al., 2017; TRIGNO CENTRO, 2024). We estimated that the sampling rate was sufficient according to a sampling theorem. To achieve life-like quality, our HMD’s refresh rate was 90 Hz, which was the same as that of the commercial VR medical device KAGURA (mediVR Inc.) (medivr, 2024). The resolution was 1440×1600 for a single eye, which was higher than that of KAGURA. Therefore, the quality was determined to be sufficient. However, the frequency of the avatar joints angle updates was dependent on the Azure Kinect DK sampling rate of 30 FPS. As mentioned above, the virtual angle was calculated within approximately 3 ms, and this calculation time was determined to be sufficient.

## 2 Methods

### 2.1 Experimental method

**FIGURE 2:**
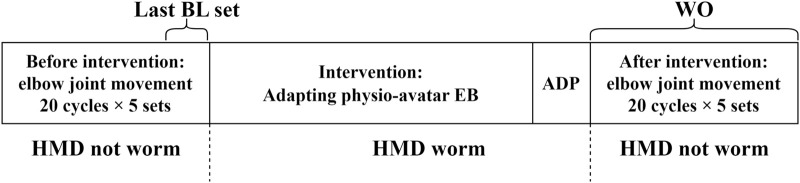
Experimental flow.

The experiments in this study, which involved human participants, were conducted with approval from the Ethics Committee for Research Involving Human Subjects at the Graduate School of Engineering Science, Osaka University (Approval number: R3-3). Intervention using the physio-avatar EB system was performed on the participants, during which the following measurements were recorded: the AA ratio, AA sum, and elbow joint angle during identical cyclic movements before the intervention, after adaptation during the physio-avatar EB intervention (ADP), and immediately after the intervention [washout (WO)] (Figure 2). The abovementioned adaptation process occurs during physio-avatar EB intervention before ADP. The HMD was only worn during the intervention, and only data recording was conducted.

**FIGURE 3:**
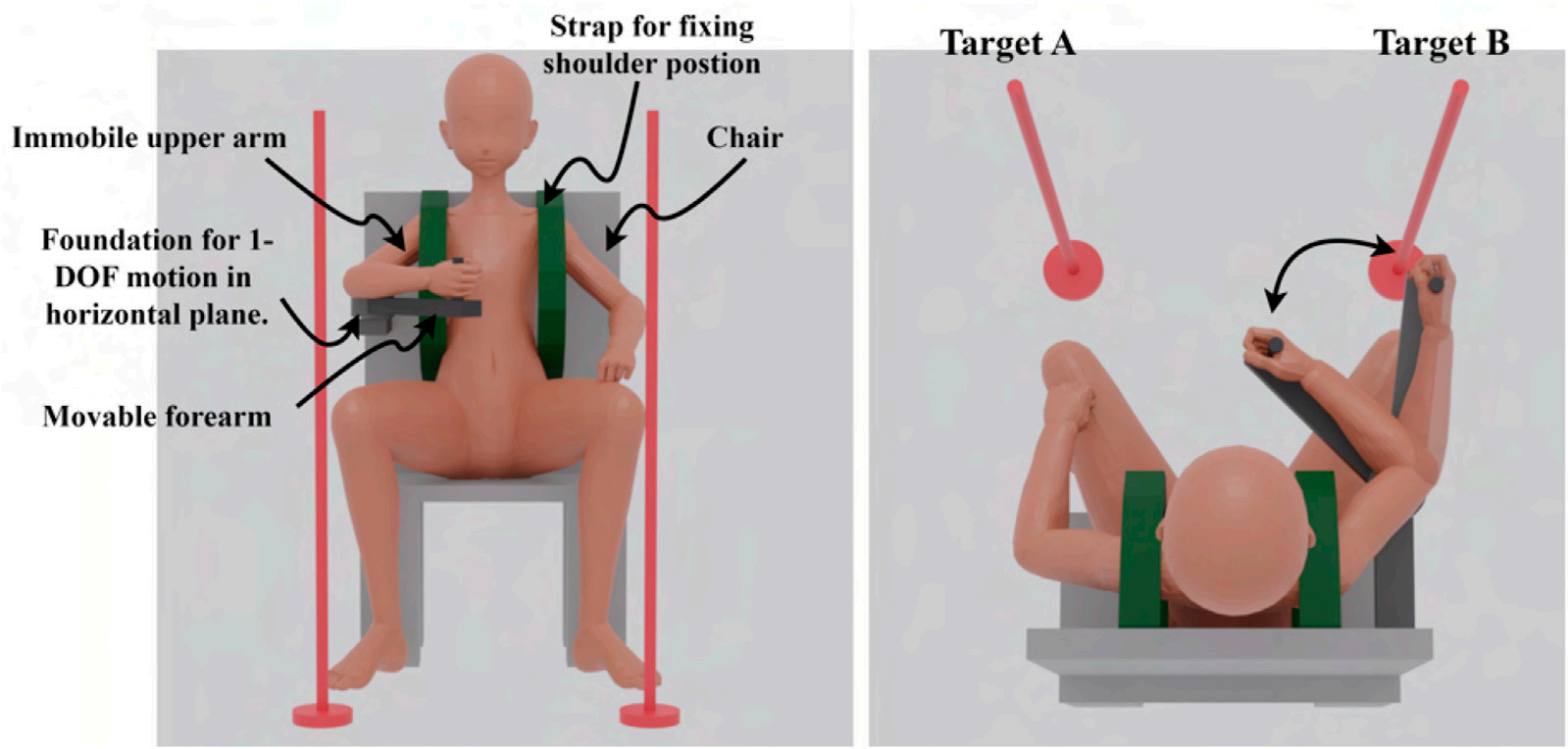
Image of the experiment without the physio-avatar EB system.

The cyclic movements before the intervention and WO were reciprocal movements performed with the eyes open. Target A was set at a point where the joint angle was 90 [deg] , and Target B was set at a point where the joint angle was 150 [deg] . Markers were placed in the real space accordingly (Figure 3). First, a sound was played to signal the start of the movement, and a sound signaling the end of the movement was played 0.9 [s] after the sound signal for the start of movement. Another sound signal for the start of movement was played 2.0 [s] after the sound signal for the end of movement. The participants repeated a 5.8 [s] cycle of extension, holding, flexion, and holding for 20 cycles as one set. This was performed in five sets during cyclic movements before the intervention and five sets during WO, with an approximately 1-min interval between sets. The “environment-adapted” motor control strategy was recorded to examine the impact on the participant’s internal model of the physio-avatar EB experience system. Therefore, five sets of cyclic movements were performed before the intervention, with the data from the fifth set serving as the pre-intervention motor control strategy [baseline (BL)]. Additionally, 20 cycles of cyclic movement were performed in five sets during WO to identify whether the physio-avatar EB intervention affected the participant’s motor control strategies.

During the adaptation process in the physio-avatar EB intervention, given the lack of a function to place markers within the VR space, the participants were instructed to remember the hand positions where the avatar’s joint angle reached 90 [deg] and 150 [deg] . Initially, the participants visually memorized these positions while being verbally guided by the experimenter, who could then check the avatar’s joint angle in real time. The adaptation assessment criteria during the physio-avatar EB intervention were as follows. The experimenter gave each participant instructions about the target angle at undetermined times. The participant was asked to control the avatar’s joint angle within ±5[deg] of the target angle. The goal was to have a success rate of 80% over the final 10 attempts. After adaptation assessment, the aforementioned cyclic movements were performed using the physio-avatar EB system to confirm the motor control strategy adapted to the avatar. This experiment involved seven participants (Participants A–G). The participants were right-handed and aged 22.5±0.9 years.

**FIGURE 4:**
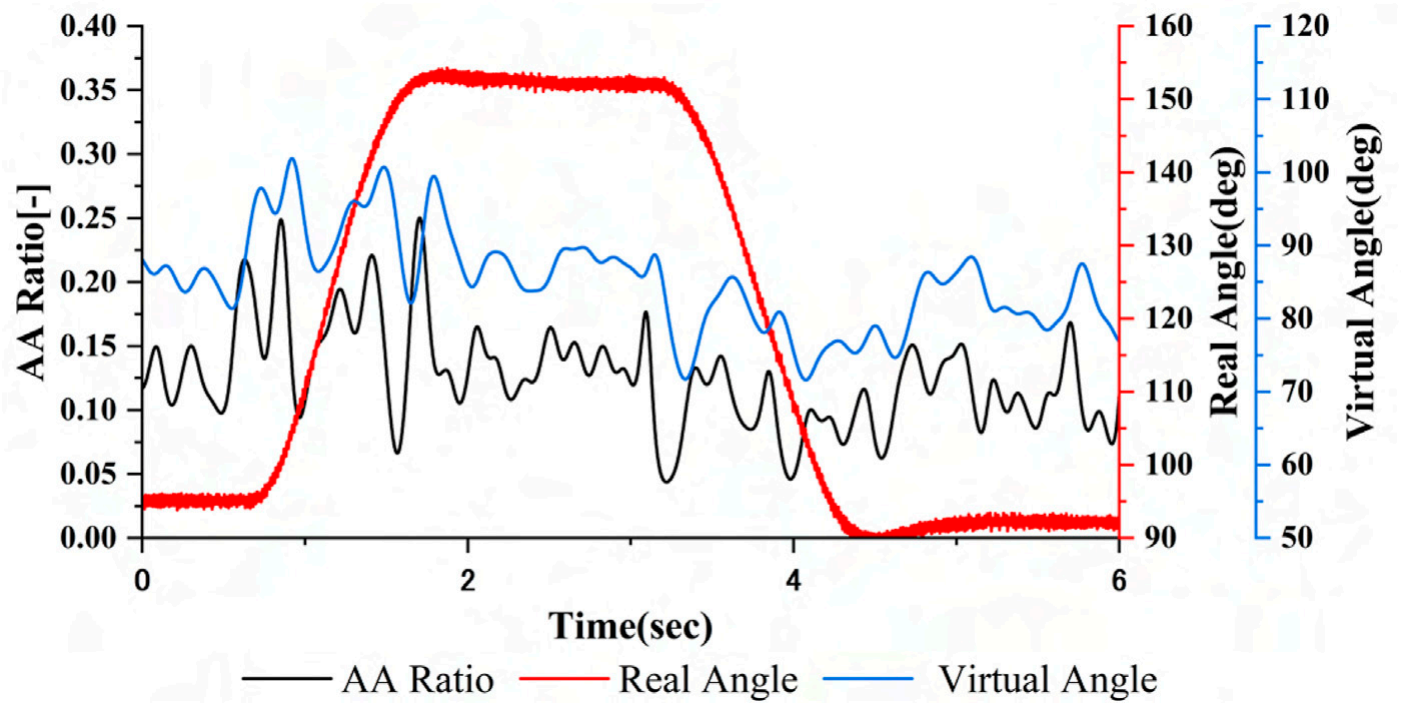
AA ratio and virtual angle calculated by the NMSS model and real angles.

All experiments were conducted with the physio-avatar EB’s NMSS model parameters, as shown in Table 1. Preliminary experiments showed that physio-avatar EB intervention with high natural frequencies of the NMSS model, namely, $\omega_{\text{nNM}}$ = 40.0, $\omega_{\text{nMS}}$ = 40.0, results in qualitatively different AA ratio trajectories before and after intervention (Ando et al., 2023; Ando et al., 2024; Ando et al., 2022). Therefore, in this experiment, the natural frequencies were set to the system’s limit values. Additionally, the damping coefficients were set to the system’s limit values to prevent excessive overshooting, which hinders control. The gain was set to a value that allowed for sufficiently perceptible extension and flexion movements of the avatar. The behavior of the physio-avatar EB characteristics is demonstrated in Figure 4, which shows the AA ratio, the virtual angle calculated using the NMSS model based on the AA ratio, and the corresponding actual joint angle. The changes in the virtual angle ahead of the real angle, qualitatively described as hyper-responsive movement, transitioned from approximately 80 [deg] to approximately 100 [deg] . This change was smaller than that of the joint angle.

**TABLE 1:** NMSS model parameters used in the study.

$K_{\text{NM}}$	$\omega_{\text{nNM}}$	$\zeta_{\text{NM}}$	$L_{\text{NM}}$	$K_{\text{MS}}$	$\omega_{\text{nMS}}$	$\zeta_{\text{MS}}$
15.0 [−]	70.0 [rad/s]	2.0 [−]	0.0 [s]	15.0 [−]	70.0 [rad/s]	2.0 [−]

**FIGURE 5:**
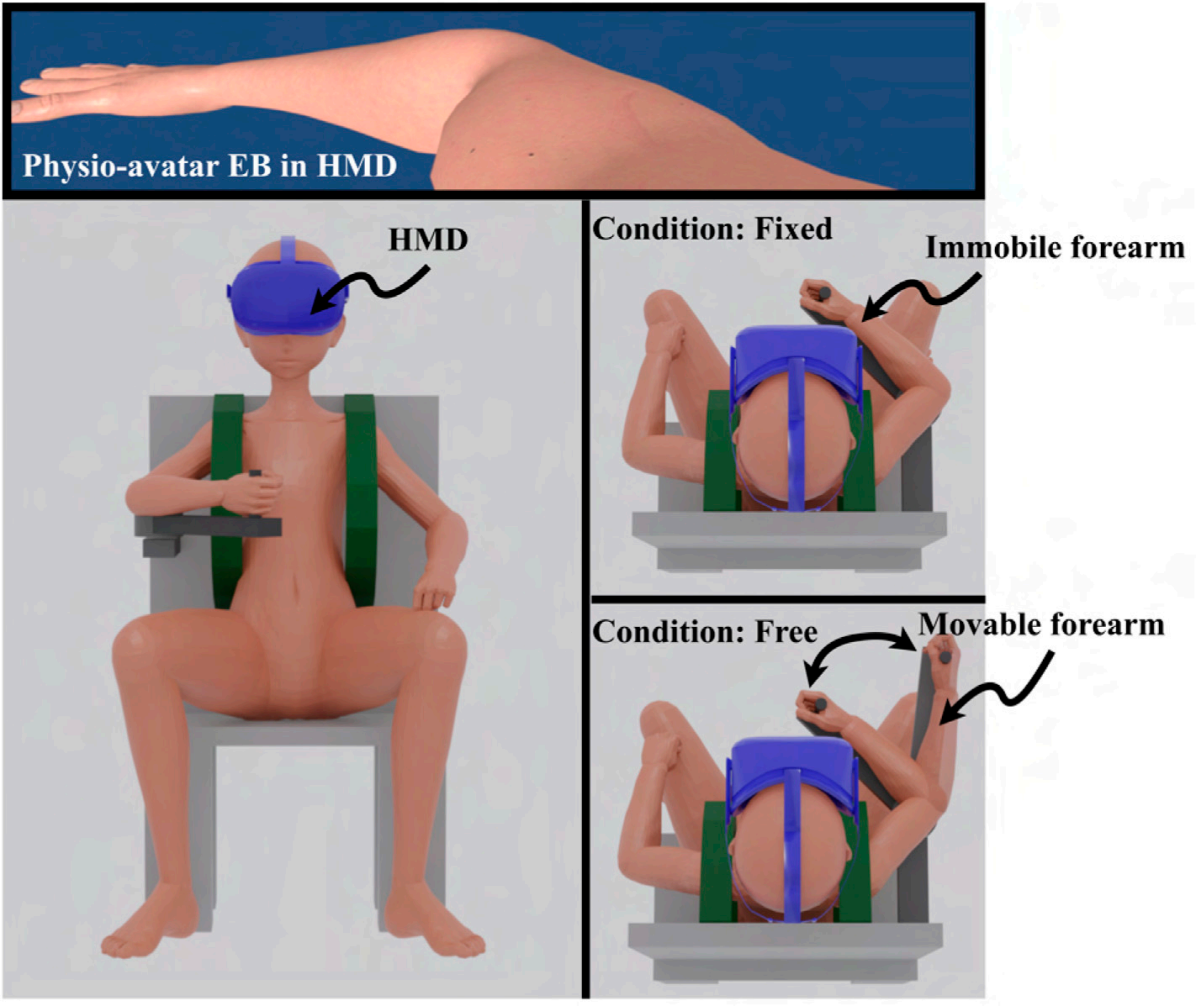
Image of the experiment using the physio-avatar EB system.

Previous studies have qualitatively demonstrated changes in motor control strategies before and after interventions when the real body’s elbow joint could extend and flex during intervention under conditions where the hand was free (Ando et al., 2023; Ando et al., 2024; Ando et al., 2022). However, the effect on motor control strategies of somatosensory input, such as counterforce, also needs to be verified, necessitating the examination of the physio-avatar intervention effect when the hand position is fixed during the intervention. Therefore, the experiments in this study were conducted under both conditions (fixed and free hand position during intervention) (Figure 5). Participants E and G only participated under the fixed-hand-position intervention condition.

**FIGURE 6:**
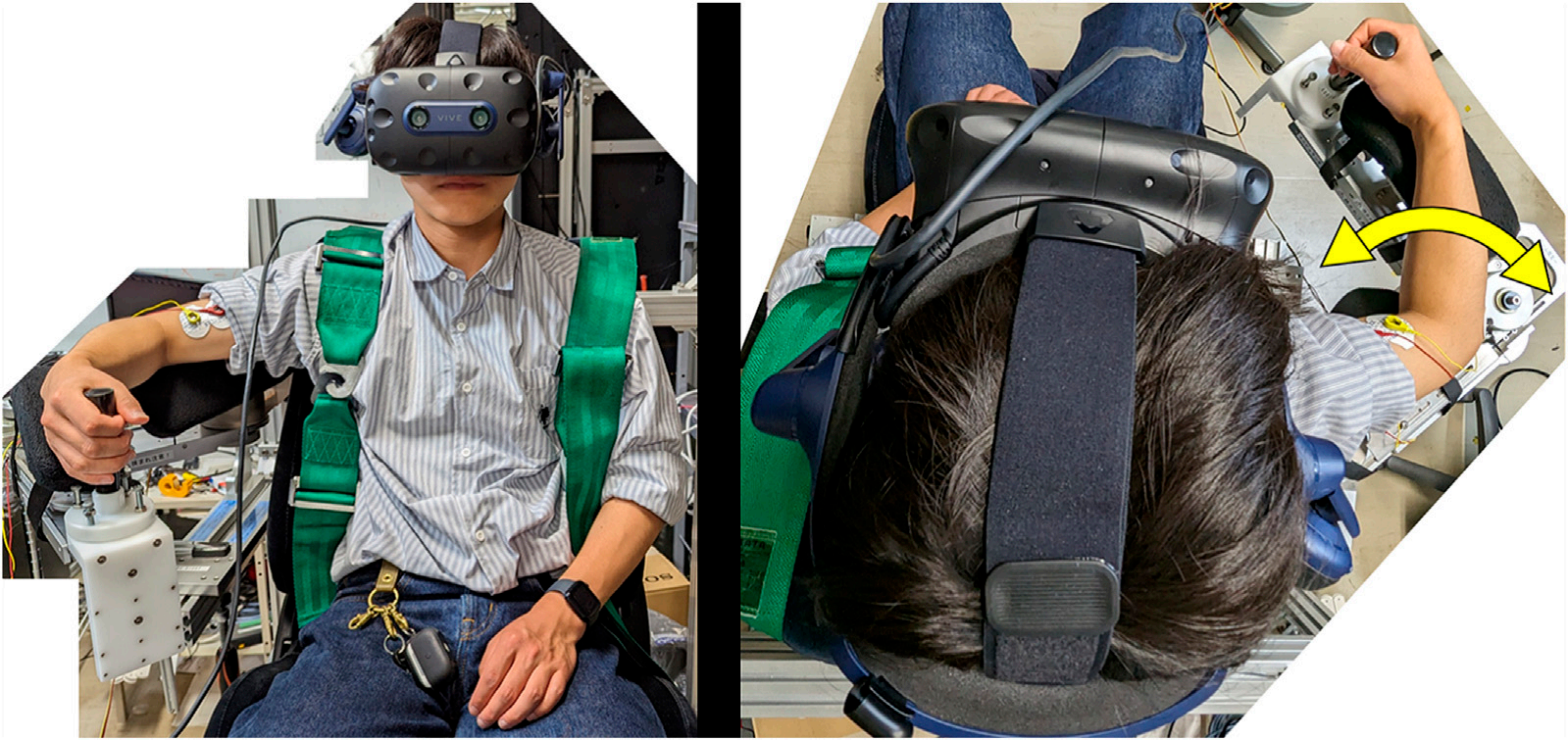
Actual images of a participant using the physio-avatar EB system (left: front view, right: top view).

In the experiment, each participant was in a sitting position. The hand rest shown in Figure 3, which had fixed and movable platforms, was used to keep the upper arm immobile and the forearm movable. This setup enabled 1-DOF motion within the horizontal plane while compensating for gravity and maintaining a constant elbow joint position. The trunk was fixed using the belt shown in Figure 3 to eliminate the influence of posture differences on motor control strategies between BL and WO. The right shoulder joint was positioned at a horizontal flexion angle of 45 [deg] , and the elbow rest on the hand rest was adjusted so that the right elbow joint was positioned 10 [cm] vertically below the right shoulder joint, and this angle was maintained. Pictures of the experiment are shown in Figure 6. A seven-point questionnaire (ranging from 1 to 7, with 1 being the lowest) regarding the sense of ownership (SoO) and sense of agency (SoA) was administered at the end of the experiment to investigate the effects of these indicators on the physio-avatar EB intervention.

### 2.2 Analysis method

The recorded AA ratio, AA sum, and joint angle (the virtual angle is used instead during ADP) were analyzed separately for the extension and flexion movements. A 1.8 [s] cycle starting 0.9 [s] before the initiation of extension, with 1800 samples per cycle, was segmented for evaluation, excluding cycles with artifacts. Although delays in movement initiation might also be affected by the intervention, the intent was to focus on the “trajectory” by considering movement initiation as the analysis subject. Hence, movement initiation was defined as when the angle during flexion or extension changed by 3.0° from a hold period during extension or flexion. During ADP, virtual angles were used instead of actual joint angles, so the movement initiation point was defined based on virtual angles. This ensured that the trajectory was evaluated based on the movement initiation of the real body or the avatar. The start of extension is marked as 0.00 [s] , and motor control strategies are evaluated from the perspective of the AA ratio trajectory, a control variable of the physio-avatar EB system.

The coefficient of determination (CoD) (square of Pearson’s product–moment correlation coefficient), an indicator of similarity between two datasets in statistics, was used for evaluation. The CoD was used to determine whether the motor control strategies were similar to those before the intervention. The waveform after averaging the BL AA ratio was defined as the BL motor control strategy. The CoD with the BL motor control strategy was calculated for all BL and WO cycles. Additionally, the average CoD for each BL cycle with respect to the BL motor control strategy was determined. The trajectories of the AA ratio and joint angle should generally align with the fact that the AA ratio approaches 1 (0) during extension (flexion); thus, the trajectory of the AA ratio should be somewhat similar for the same movement. Therefore, an excessively low average CoD for the BL motor control strategy for each BL cycle indicated possible issues with EMG acquisition, such as insufficient signal levels or noise. A threshold value of 0.25 was set for the average CoD, and evaluations were performed on experimental results where the average CoD of the BL motor control strategy for each BL cycle during flexion and extension exceeded 0.25. Although this threshold lacked a clear basis, it was set according to a previously published guideline that a “correlation coefficient up to 0.5 is considered sufficient” (Akoglu, 2018).

In the learning of the internal model, systems are used for the short- and long-term memory of motor control strategies (Osu et al., 2002). Time constants are used to explain the results of these memories, so the time constants in this study were determined by performing exponential approximation on all 100 WO cycles. The approximate exponential function is shown in Equation 5.

$$y=a\left(1-e^{-\left(x-1\right)/b}\right)+c$$ (5)

a , b , and c are constants. y represents the CoD for each cycle, and x denotes the number of flexion and extension movement cycles during WO. a is the difference between the convergence value and the initial value of the approximate exponential function, b is the time constant, and c represents the initial value of the approximate exponential function. The exponential approximation curve was calculated using Origin 2021 version 9.80 (LightStone Corp.). In the calculation process, the function model was set as an explicit function, and the function form was set as an arithmetic expression. The initial values of a , b , and c were set to 1, and no boundary conditions were set. The calculation yielded the estimated values of each parameter for the fit that created the curve closest to the data points and the standard error of the parameters representing the accuracy of the approximation curve. A small standard error indicated a high accuracy of the approximation curve, whereas a large standard error suggested that the exponential approximation did not fit the data well. Furthermore, if the exponential approximation curve did not converge and became a straight line, the control strategy had already converged at the start of WO, or the exponential approximation did not fit the data well (OriginLab, 2023). If the standard error of each parameter of the exponential approximation curve was larger than the estimated values or if the exponential approximation curve did not converge, then the data were regarded as experimental data that did not fit the exponential approximation. The waveform after averaging the AA ratio during ADP was defined as the ADP motor control strategy. The CoD of the ADP motor control strategy was calculated for all ADP and WO cycles. As for the CoD of the ADP, the time constant was determined by performing exponential approximation targeting all WO cycles. Exponential approximation could be conducted similarly to that for the CoD of the BL motor control strategy during WO. Additionally, considering the possibility of deviation from the motor control strategy adapted to the avatar, the initial value of a was changed to −1 for the calculation of the exponential approximation curve.

Because the AA sum was not a control variable during the physio-avatar EB intervention, the motor control strategy appeared not to change due to the physio-avatar EB intervention. However, studies on variability between movement cycles state that the variability of task-related control variables decreases, whereas the variability of non-task-related control variables does not decrease (Dal’Bello and Izawa, 2021). Human movement involves control variables, such as the equilibrium point and stiffness. During adaptation to the physio-avatar EB system, that equilibrium point is a task-related control variable, and stiffness is a non-task-related control variable. The variability of indicators related to a control variable (equilibrium point) may decrease, whereas the variability of indicators related to a non-control variable (stiffness) may increase. The waveform of the AA ratio was evaluated for the equilibrium point; for stiffness, the total sum of all samples of the AA sum was utilized as an evaluation index for BL and ADP. For the equilibrium point, the standard deviation of each sample of the AA ratio across all cycles was calculated during BL and ADP, and its average value served as an indicator of variability. As for stiffness, EMG variations increase at high intensities of muscle activity; the variation of the AA sum, which is the sum of the EMG signals, also increases at high intensities (Gasparic et al., 2023). Therefore, the coefficient of variation was utilized as an indicator of variability; the standard deviation of the total sum of the AA sum was normalized across all cycles using the average value for BL and ADP.

The variability indicators for the equilibrium point in BL and ADP were denoted as $\sigma_{\text{BR}}$ and $\sigma_{\text{AR}}$ , respectively, and the variability indicators for stiffness in BL and ADP were $\sigma_{\text{BS}}$ and $\sigma_{\text{AS}}$ , respectively. As for the increase in variability from BL, the variation of the equilibrium point index was calculated by dividing $\sigma_{\text{AR}}$ by $\sigma_{\text{BR}}$ , and the variation of the stiffness index was calculated by dividing $\sigma_{\text{AS}}$ by $\sigma_{\text{BS}}$ . Let i be the cycle number and j be the sample number. The methods for calculating the total sum of the AA sum for all samples, the variability index for the equilibrium point, and the variability index for stiffness are shown in Equations 6–8, respectively.

$$S_{i}=\overset{1800}{\underset{j=1}{\sum }}s_{i,j}.$$ (6)

$$\sigma_{\text{BR}}=\frac{\overset{1800}{\underset{j=1}{\sum }}\sqrt{\frac{1}{20}\overset{20}{\underset{i=1}{\sum }}{\left(R_{i,j}-\bar{R_{j}}\right)}^{2}}}{1800}.$$ (7)

$$\sigma_{\text{BS}}=\frac{\sqrt{\frac{1}{20}\overset{20}{\underset{i=1}{\sum }}{\left(S_{i}-\bar{S}\right)}^{2}}}{\bar{S}}.$$ (8)

$s_{i,j}$ is the AA sum for each cycle and sample, $S_{i}$ is the stiffness for each cycle, $R_{i,j}$ is the AA ratio for each cycle and sample, $\bar{R_{j}}$ is the average value of the AA ratio for each sample across all cycles, and $\bar{S}$ is the average value of stiffness for all cycles. When adapted to the physio-avatar EB system, the variability of the equilibrium point, a control variable, should decrease compared with the variability of stiffness, a non-control variable (Dal’Bello and Izawa, 2021). Therefore, the degree of adaptation to the physio-avatar EB system was evaluated by comparing $\Delta \sigma_{\mathrm{AAR}}$ and $\Delta \sigma_{St}$ .

Finally, whether the same movements were being performed during the BL and WO periods should be confirmed during the evaluation of the changes in motor control strategies due to avatar intervention. As in the analysis of the AA ratio, the waveform after averaging the joint angle was defined as the BL joint angle, and the CoD of the joint angle for all WO cycles was calculated. The trajectory of the joint angle had small errors between cycles. Therefore, only cycles where the CoD of the BL joint angle exceeded 0.95 were determined to include movements similar to the BL ones.

## 3 Results

The following indices are used to discuss the experimental results for both flexion and extension movements.

• Average CoD at BL (Avg CoD (BL)): the average CoD for each BL cycle against the AA ratio trajectory during the BL motor control strategy

• Standard deviation CoD at BL (SD of CoD (BL)): the standard deviation of the CoD for each BL cycle against the AA ratio trajectory during the BL motor control strategy

• Time constant (BL): the time constant of the exponential function approximating the CoD against the AA ratio trajectory during the BL motor control strategy during WO

• Initial Value (BL): the initial value of the exponential function approximating the CoD against the AA ratio trajectory during the BL motor control strategy during WO

• Constant Value (BL): the convergence value of the exponential function approximating the CoD against the AA ratio trajectory during the BL motor control strategy during WO

• $\sigma_{\text{BR}}$ : variability index of the equilibrium point at BL

• $\sigma_{\text{BS}}$ : variability index of stiffness at BL

• Average CoD at ADP (Avg of CoD (ADP)): the average CoD for each ADP cycle against the AA ratio trajectory during the ADP motor control strategy

• Standard deviation of CoD at ADP (SD of CoD (ADP)): the standard deviation of the CoD for each ADP cycle against the AA ratio trajectory during the ADP motor control strategy

• Time Constant (ADP): the time constant of the exponential function approximating the CoD against the AA ratio trajectory during the ADP motor control strategy during WO

• Initial Value (ADP): the initial value of the exponential function approximating the CoD against the AA ratio trajectory during the ADP motor control strategy during WO

• Constant Value (ADP): the convergence value of the exponential function approximating the CoD against the AA ratio trajectory during the ADP motor control strategy during WO

• $\sigma_{\text{AR}}$ : variability index of the equilibrium point during ADP

• $\sigma_{\text{AS}}$ : variability index of stiffness during ADP

• Variation of equilibrium point index during flexion (VEPFX): the variability increase of the equilibrium point during flexion in the ADP period relative to the BL period

• Variation of stiffness index during flexion (VSTFX): the variability increase of stiffness during flexion in the ADP period relative to the BL period

• Variation of equilibrium point index during extension (VEPEX): The variability increase of the equilibrium point during extension in the ADP period relative to the BL period

• Variation of stiffness index during extension (VSTEX): the variability increase of stiffness during extension in the ADP period relative to the BL period

• CoD of the angle during flexion (CoD Angle (FX)): the CoD against the averaged joint angle trajectory at BL during the first cycle of the first set in the WO period during flexion

• CoD of the angle during extension (CoD Angle (EX)): the CoD against the averaged joint angle trajectory at BL during the first cycle of the first set in the WO period during extension

• SoA: sense of agency

• SoO: sense of ownership

For CoD Angle (FX) and CoD Angle (EX), if the value of the first cycle is small, then the value of the second cycle is shown in parentheses. Additionally, experimental data that do not fit the exponential approximation are denoted with a “-” for the time constant. In that case, the Initial Value (BL) or Initial Value (ADP) and Constant Value (BL) or Constant Value (ADP) are shown as average values during WO. Additionally, for Participants C, D, E, and G under the Free condition and Participant F under both conditions, the average CoD values at BL are below 0.25, leading to their exclusion from evaluation. Thus, the experimental results considered for evaluation are those of Participants A and B under both conditions and those of Participants C, D, and E under the Fixed condition. The results are shown in Tables 2–6.

**TABLE 2:** Results: VEPFX, VSTFX, VEPEX, VSTEX, CoD angle (FX), CoD angle (EX), SoA, and SoO.

	VEPFX	VSTFX	VEPEX	VSTEX	CoD angle (FX)	CoD angle (EX)	SoA	SoO
A (Fixed)	1.946	2.435	1.694	3.237	0.996	0.979	3	2
A (Free)	1.069	1.275	1.035	1.867	0.992	0.995	3	3
B (Fixed)	0.901	2.566	0.738	2.124	0.999	0.688 (0.995)	4	5
B (Free)	1.013	3.004	0.741	1.943	0.999	0.999	3	3
C	1.354	3.189	0.917	1.883	0.999	0.99	6	2
D	1.354	2.066	0.759	2.098	0.999	0.886 (0.988)	3	4
E	4.130	3.226	2.054	5.981	0.995	0.534 (0.996)	5	4

**TABLE 3:** Results during flexion movement: Avg of CoD (BL), SD of CoD (BL), Time Constant (BL), Initial Value (BL), Constant Value (BL), $\sigma_{\text{BR}}$ , and $\sigma_{\text{BS}}$ .

	Avg of CoD (BL)	SD of CoD (BL)	Time constant (BL)	Initial value (BL)	Constant value (BL)	$\sigma_{\text{BR}}$	$\sigma_{\text{BS}}$
A (Fixed)	0.791	0.051	-	0.711	0.711	0.074	0.108
A (Free)	0.818	0.041	-	0.767	0.767	0.105	0.106
B (Fixed)	0.516	0.114	14.786	0.061	0.607	0.136	0.145
B (Free)	0.518	0.156	-	0.531	0.531	0.130	0.196
C	0.677	0.080	8.480	0.415	0.657	0.097	0.073
D	0.622	0.120	-	0.540	0.540	0.111	0.229
E	0.617	0.131	15.548	0.249	0.488	0.023	0.066

**TABLE 4:** Result during extension movement: Avg of CoD (BL), SD of CoD (BL), Time Constant (BL), Initial Value (BL), Constant Value (BL), $\sigma_{\text{BR}}$ , and $\sigma_{\text{BS}}$ .

	Avg of CoD (BL)	SD of CoD (BL)	Time constant (BL)	Initial value (BL)	Constant value (BL)	$\sigma_{\text{BR}}$	$\sigma_{\text{BS}}$
A (Fixed)	0.775	0.097	-	0.725	0.725	0.085	0.076
A (Free)	0.824	0.075	-	0.796	0.796	0.107	0.089
B (Fixed)	0.348	0.150	8.803	0.051	0.392	0.133	0.213
B (Free)	0.377	0.188	49.122	0.542	0.232	0.146	0.193
C	0.600	0.098	20.171	0.430	0.592	0.121	0.093
D	0.516	0.114	-	0.553	0.553	0.143	0.171
E	0.523	0.157	5.842	0.128	0.477	0.037	0.043

**TABLE 5:** Results during flexion movement: Avg of CoD (ADP), SD of CoD (ADP), Time Constant (ADP), Initial Value (ADP), Constant Value (ADP), $\sigma_{\text{AR}}$ , and $\sigma_{\text{AS}}$ .

	Avg of CoD (ADP)	SD of CoD (ADP)	Time constant (ADP)	Initial value (ADP)	Constant value (ADP)	$\sigma_{\text{AR}}$	$\sigma_{\text{AS}}$
A (Fixed)	0.358	0.138	-	0.451	0.451	0.144	0.263
A (Free)	0.380	0.150	-	0.319	0.319	0.112	0.135
B (Fixed)	0.549	0.181	-	0.214	0.214	0.122	0.372
B (Free)	0.451	0.133	49.285	0.458	0.205	0.132	0.588
C	0.670	0.163	-	0.503	0.503	0.132	0.234
D	0.631	0.140	-	0.309	0.309	0.150	0.473
E	0.455	0.147	-	0.283	0.283	0.095	0.213

**TABLE 6:** Results during extension movement: Avg of CoD (ADP), SD of CoD (ADP), Time Constant (ADP), Initial Value (ADP), Constant Value (ADP), $\sigma_{\text{AR}}$ and $\sigma_{\text{AS}}$

	Avg of CoD (ADP)	SD of CoD (ADP)	Time constant (ADP)	Initial value (ADP)	Constant value (ADP)	$\sigma_{\text{AR}}$	$\sigma_{\text{AS}}$
A (Fixed)	0.563	0.211	-	0.595	0.595	0.144	0.246
A (Free)	0.540	0.158	-	0.707	0.707	0.111	0.167
B (Fixed)	0.804	0.081	-	0.246	0.246	0.098	0.453
B (Free)	0.518	0.137	35.115	0.480	0.184	0.108	0.375
C	0.623	0.136	-	0.267	0.267	0.111	0.175
D	0.632	0.168	-	0.372	0.372	0.109	0.358
E	0.732	0.127	-	0.344	0.344	0.076	0.255

**FIGURE 7:**
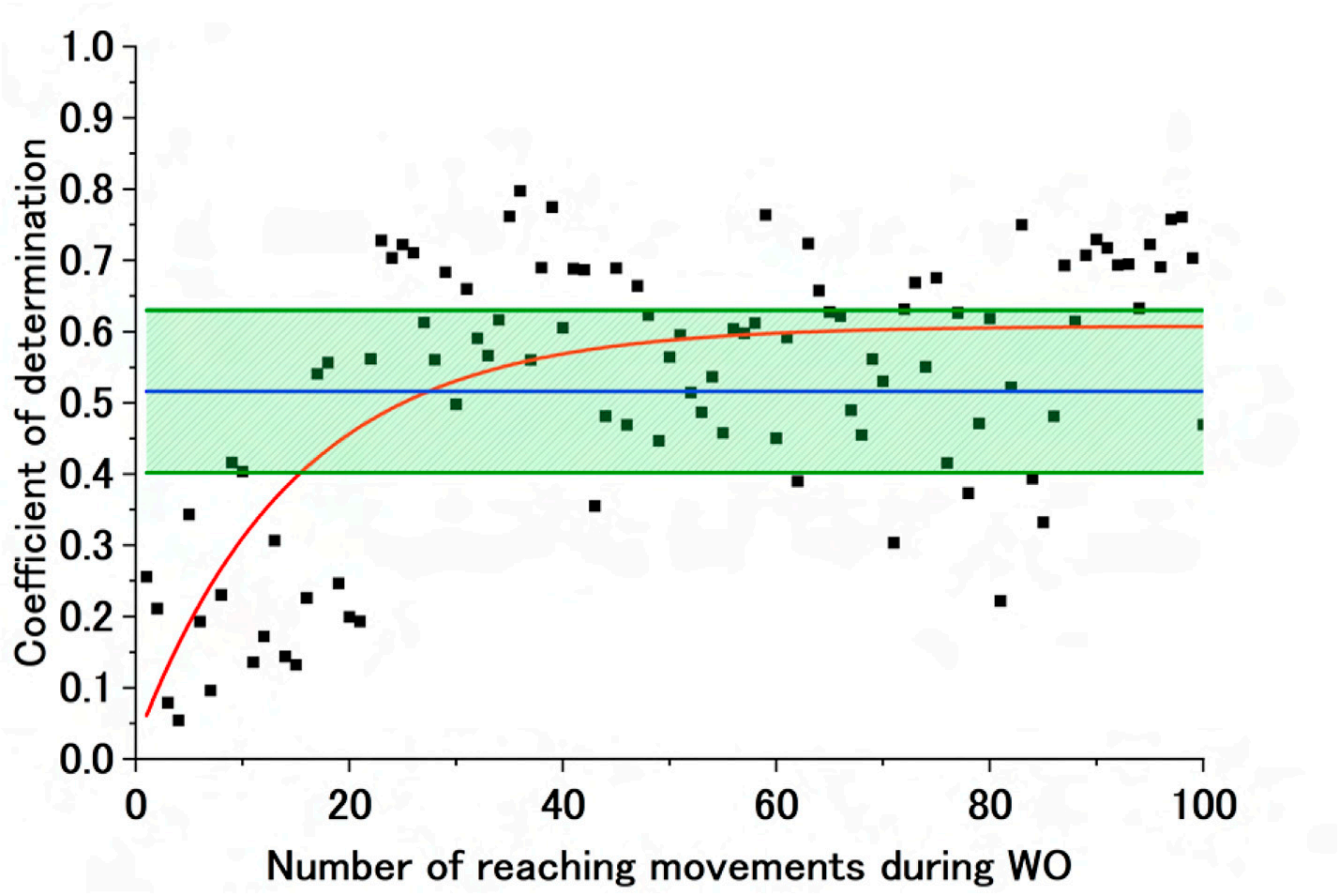
Sample exponential approximation curve (calculated) of the CoD of the BL motor control strategy during WO. The black dots represent the CoD of the BL motor control strategy, and the red line represents the failed approximated exponential function of the CoD of the BL motor control strategy. The blue line represents Avg of CoD (BL), and the green lines represent Avg of CoD (BL) + SD of CoD (BL) and Avg of CoD (BL) − SD of CoD (BL).

**FIGURE 8:**
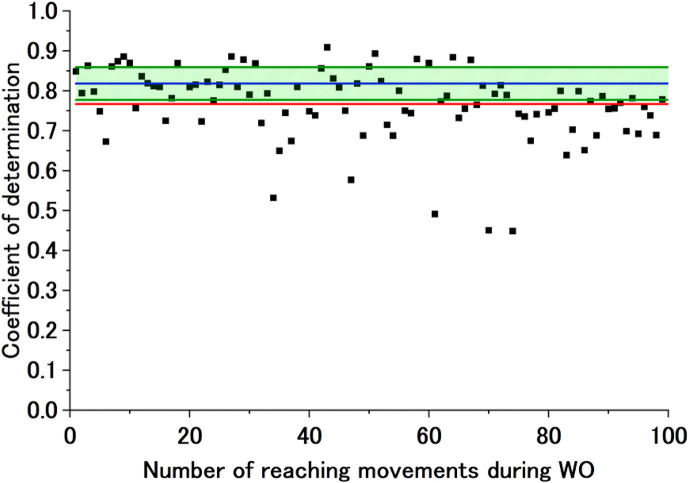
Sample exponential approximation curve (not calculated) of the CoD of the BL motor control strategy during WO. The black dots represent the CoD of the BL motor control strategy, and the red line represents the failed approximated exponential function of the CoD of the BL motor control strategy. The blue line represents Avg of CoD (BL), and the green lines represent Avg of CoD (BL) + SD of CoD (BL) and Avg of CoD (BL) − SD of CoD (BL).

Figure 7 shows sample experimental results under a converged exponential approximation curve of the CoD of the BL motor control strategy during WO. These are the results during flexion for Participant B (Fixed). The graph shows the CoD of the AA ratio of the BL motor control strategy during WO, the exponential approximation curve calculated from that CoD, Avg of CoD (BL), and Avg of CoD (BL) ± SD of CoD (BL). The exponential approximation curve during WO starts sufficiently lower than Avg of CoD (BL). Subsequently, it converges within the range of Avg of CoD (BL) ± SD of CoD (BL), reaching a value equivalent to Avg of CoD (BL). Initially, a motor control strategy different from that at BL is maintained, but the BL motor control strategy dominates again as the cycles increase. Figure 8 shows sample experimental results for a case where the data of the CoD of the BL motor control strategy during WO do not fit the exponential approximation. These are the results during flexion for Participant A (Free). The graph shows the CoD of the AA ratio of the BL motor control strategy during WO, the failed exponential approximation curve calculated from that CoD, Avg of CoD (BL), and Avg of CoD (BL) ± SD of CoD (BL). The failed exponential approximation curve during WO is outside the range of Avg of CoD (BL) ± SD of CoD (BL) but is equivalent to Avg of CoD (BL) − SD of CoD (BL). Additionally, cycles above Avg of CoD (BL) can be observed from the early WO period, indicating a return to the BL motor control strategy immediately after the end of the physio-avatar EB intervention.

**FIGURE 9:**
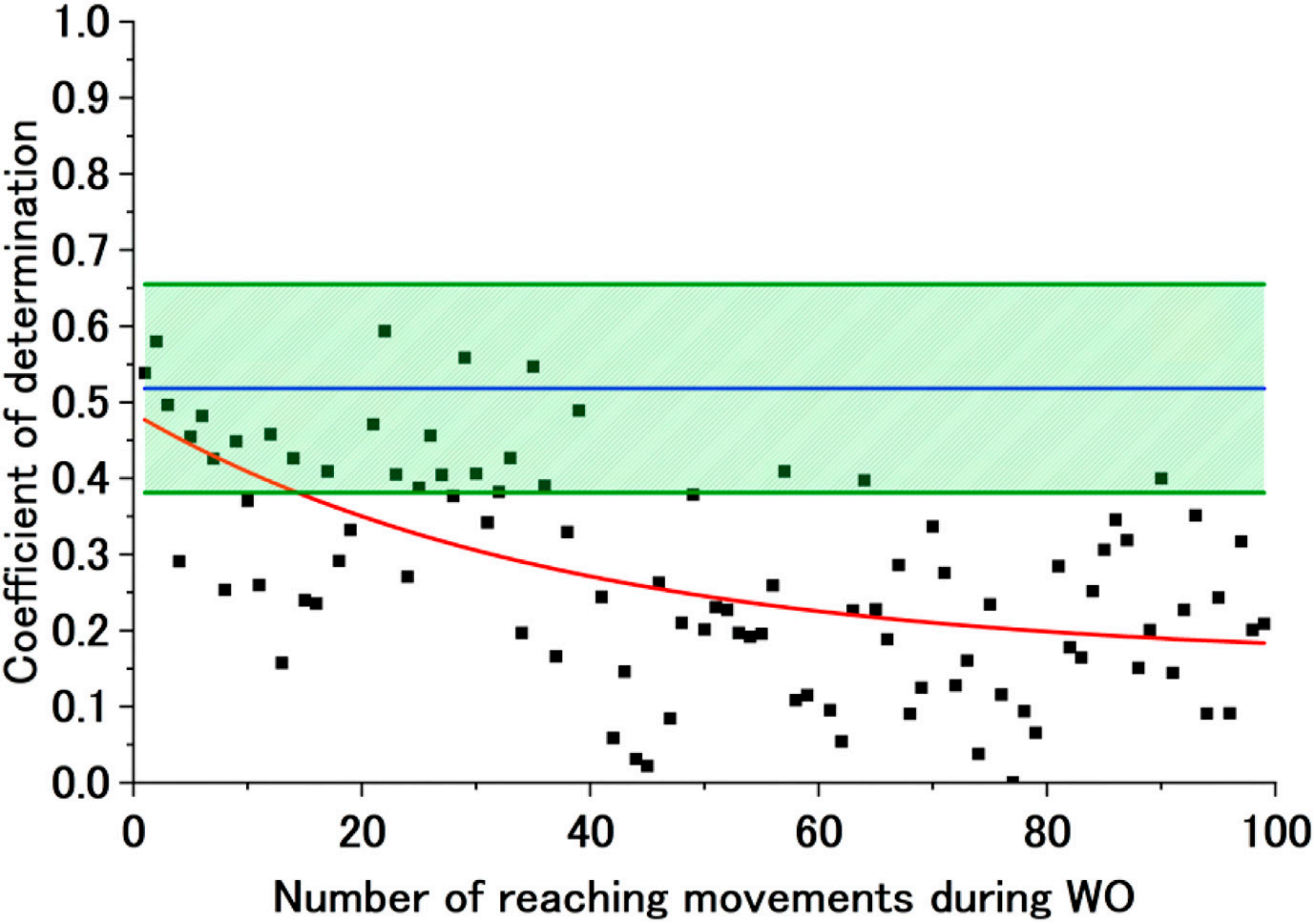
Sample exponential approximation curve (calculated) of the CoD of the ADP motor control strategy during WO. The black dots represent the CoD of the ADP motor control strategy, and the red line represents the failed approximated exponential function of the CoD of the ADP motor control strategy. The blue line represents Avg of CoD (ADP), and the green lines represent Avg of CoD (ADP) + SD of CoD (ADP) and Avg of CoD (ADP) − SD of CoD (ADP).

**FIGURE 10:**
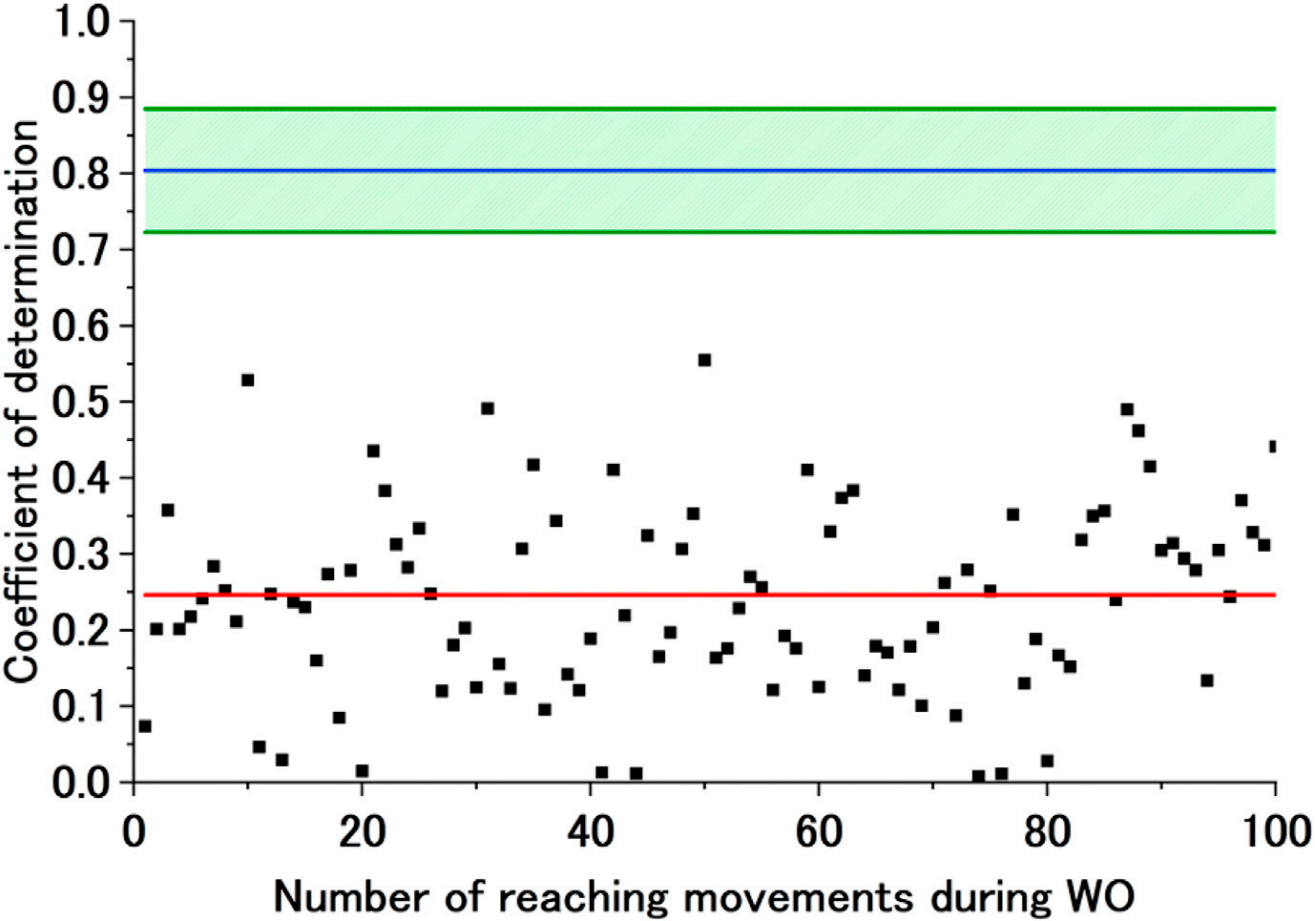
Sample exponential approximation curve (not calculated) of the CoD of the ADP motor control strategy during WO. The black dots represent the CoD of the ADP motor control strategy, and the red line represents the failed approximated exponential function of the CoD of the ADP motor control strategy. The blue line represents Avg of CoD (ADP), and the green lines represent Avg of CoD (ADP) + SD of CoD (ADP) and Avg of CoD (ADP) − SD of CoD (ADP).

Figure 9 shows sample experimental results under a converged exponential approximation curve of the CoD of the ADP motor control strategy during WO. These are the results during extension for Participant B (Free). The graph shows the CoD of the AA ratio of the ADP motor control strategy during WO, the exponential approximation curve calculated from that CoD, and Avg of CoD (ADP) ± SD of CoD (ADP). The exponential approximation curve of the CoD in the early WO period is within the range of Avg of CoD (ADP) ± SD of CoD (ADP), but it is outside the range in the following phase. Initially, a motor control strategy similar to the ADP strategy is maintained, but it diverges from the ADP motor control strategy as the experiment progresses to the later phases. Figure 10 shows sample experimental results where the exponential approximation curve of the CoD of the ADP motor control strategy during WO does not fit the exponential approximation. These results are for Participant B (Fixed). The graph shows the CoD of the AA ratio of the ADP motor control strategy during WO, the failed exponential approximation curve calculated from that CoD, and Avg of CoD (ADP) ± SD of CoD (ADP). The CoD of the ADP motor control strategy during WO starts with values sufficiently lower than the CoD during ADP from the outset, indicating a deviation from the ADP motor control strategy immediately after the end of the physio-avatar EB intervention.

Table 2 shows VEPFX, VEPEX, VSTFX, VSTEX, CoD angle (FX), and CoD angle (EX) in the first (or second) cycle of the first set during WO, along with SoA and SoO. Table 3 shows the indicators (Avg of CoD (BL), SD of CoD (BL), Time Constant (BL), Initial Value (BL), and Constant Value (BL)), $\sigma_{\text{BR}}$ , and $\sigma_{\text{BS}}$ for the motor control strategy during flexion at BL. Similarly, Table 4 shows the indicators (Avg of CoD (BL), SD of CoD (BL), Time Constant (BL), Initial Value (BL), and Constant Value (BL)), $\sigma_{\text{BR}}$ , and $\sigma_{\text{BS}}$ for the motor control strategy during extension at BL. In Table 3, 4, except the values for the flexion movements of Participant E, all VSTFX and VSTEX values exceed VEPEX and VEPFX, respectively, indicating suppression of the variability of the equilibrium point, a control variable during physio-avatar EB control. Additionally, the $\sigma_{\text{BR}}$ value of Participant E during flexion is the smallest among all participants, as seen in Table 3. Thus, the variability of the equilibrium point of Participant E is small, and VFPFX excessively increases during the process of dividing the $\sigma_{\text{BR}}$ value. In Dal’Bello and Izawa (2021), control variables had lower variability than non-control variables, which is similar to this outcome, showing adaptation to the physio-avatar EB system in all but one experiment. Moreover, the CoD Angle (FX) and CoD Angle (EX) during WO exceed 0.95 in the second cycle across all data and remain above 0.95 in the subsequent cycles, indicating an early return to the BL joint movement. The SoA is reported as 4 or higher (which is relatively high) by Participants B (Fixed), C, and E. Similarly, Participants B (Fixed), D, and E report SoO values of 4 or higher. However, the surveys conducted with Participants A and B reveal different SoA and SoO outcomes, depending on the hand condition.

The Time Constant (BL) and Time Constant (ADP) values of Participants B (Fixed), C, and E are calculated through exponential approximation for both flexion and extension movements. Although the Initial Values (BL) are outside the range of Avg of CoD (BL) ± SD of CoD (BL), the Constant Values (BL) converge within the range of Avg of CoD (BL) ± SD of CoD (BL). Therefore, despite initially deviating from the BL motor control strategy, the participants return to the BL motor control strategy toward the later stages of WO. The Time Constant (BL) values of Participant A under both conditions and Participant D are not calculated, indicating the experimental data do not fit the exponential approximation. For extension movements under both conditions for Participant A and for both flexion and extension movements for Participant D, the Constant Values (BL) fall within the range of Avg of CoD (BL) ± SD of CoD (BL), indicating early convergence to the BL motor control strategy. However, for flexion movements under both conditions for Participant A, the Constant Value (BL) is outside the range of Avg of CoD (BL) ± SD of CoD (BL). The data of Participant B (Free) do not fit the exponential approximation dataset, so their Time Constant (BL) value during flexion is not calculated. Constant Value (BL) falls within the range of Avg of CoD (BL) ± SD of CoD (BL), suggesting an early return to the BL motor control strategy from the start of WO. However, during extension movements, Time Constant (BL) is calculated through exponential approximation, and Constant Value (BL) converges within the range of Avg of CoD (BL) ± SD of CoD (BL); Initial Value (BL) is higher than Constant Value (BL) and Avg of CoD (BL) + SD of CoD (BL). Despite Participants B (Fixed), C, and E returning to the BL motor control strategy from a different motor control strategy, the extension movements of Participant B (Free) show convergence in the direction deviating from the BL motor control strategy, resulting in a unique outcome.

Table 5 shows the indicators (Avg of CoD (ADP), SD of CoD (ADP), Time Constant (ADP), Initial Value (ADP), and Constant Value (ADP)), $\sigma_{\text{AR}}$ , and $\sigma_{\text{AS}}$ for the ADP motor control strategy during flexion. Table 6 shows the indicators (Avg of CoD (ADP), SD of CoD (ADP), Time Constant (ADP), Initial Value (ADP), and Constant Value (ADP)), $\sigma_{\text{AR}}$ , and $\sigma_{\text{AS}}$ for the ADP motor control strategy during extension. The Time Constant (ADP) value of Participant B (Free) is calculated through exponential approximation for both flexion and extension movements. Initial Value (ADP) is within the range of Avg of CoD (ADP) ± SD of CoD (ADP), but Constant Value (ADP) is outside the range of Avg of CoD (ADP) ± SD of CoD (ADP). Thus, although the ADP motor control strategy is maintained in the early stages of WO, the participant deviates from the ADP motor control strategy in the later stages. Additionally, for the flexion movements of Participant A under both conditions and for the extension movements of Participant A (Fixed), Constant Value (ADP) falls within the range of Avg of CoD (ADP) ± SD of CoD (ADP), suggesting the possibility that the ADP motor control strategy is maintained throughout WO. Furthermore, during the extension movements of Participant A (Free), Constant Value (ADP) exceeds Avg of CoD (ADP) + SD of CoD (ADP), indicating that the ADP motor control strategy may be maintained throughout WO. For Participants B (Fixed), C, D, and E, during both flexion and extension movements, Constant Value (ADP) is outside the range of Avg of CoD (ADP) ± SD of CoD (ADP), showing a deviation from the ADP motor control strategy from the beginning of WO. Participants B (Fixed), C, and E return to the BL motor control strategy from a different motor control strategy during WO. Additionally, convergence in a direction deviating from the BL motor control strategy is observed for Participant B (Free) during extension movements. Furthermore, this participant deviates from the ADP motor control strategy during both flexion and extension movements.

## 4 Discussion

Adaptation to the physio-avatar EB system was demonstrated in six of seven experiments, indicating that the trajectory of the joint angles during flexion and extension movements returned to the BL movement strategy by the second cycle of the first set during WO. Additionally, the results of the SoA and SoO surveys in response to the physio-avatar EB system were presented. Adaptation to the avatar was confirmed by the reduced variability in control variables compared with the non-control variables. Hence, humans may be able to control variables independently, such as the equilibrium point (control variable) and stiffness (non-control variable). Moreover, the WO motor control strategy was analyzed against the BL and ADP strategies using the CoD. The (1) presence or absence of aftereffects due to the physio-avatar EB intervention and (2) its impact on SoA and SoO aftereffects were demonstrated. First, the existence of aftereffects from the WO strategy compared to the BL strategy was examined. The purpose of this study was to verify whether physio-avatar EB intervention could influence motor control strategies, so BL and WO movements had to be equivalent to evaluate the same motor control strategies. Therefore, as an indicator of the occurrence of aftereffects, the time constant of the exponential approximation of the CoD of the AA ratio trajectory during WO must include at least two cycles.

For Participants B (Fixed), C, and E during both flexion and extension movements and Participant B (Free) during extension movements, the time constant of exponential approximation was greater than 2. In these experiments, Constant Value (BL) was within the range of Avg of CoD (BL) ± SD of CoD (BL). However, for Participants B (Fixed), C, and E, Constant Value (BL) exceeded Initial Value (BL); for Participant B (Free) during extension movements, Constant Value (BL) was smaller than Initial Value (BL). The Time Constant (BL) value (larger than 2) and the return to the BL motor control strategy toward the later stages of WO suggest that aftereffects occurred due to the intervention. During the extension movements of Participant B (Free), Avg of CoD (BL) was comparatively low. The AA ratio’s trajectory at BL was averaged across cycles, resulting in a smoothed trajectory. The averaged BL motion control strategy (i.e., the averaged AA ratio trajectory) was expressed similarly to virtual trajectory control in which “the motion command is similar to the shape of the hand trajectory” (Bizzi et al., 1984). The low Avg of CoD (BL) suggests that the AA ratio’s trajectory for each BL cycle deviated from the virtual trajectory similar to the averaged trajectory of the AA ratio at BL. The deviation of a control strategy from the virtual-trajectory-like AA ratio at BL was recognized due to the real body’s stiffness being a control variable. However, in the physio-avatar EB system, only the AA ratio is a control variable, and a non-virtual-trajectory-control-like control strategy cannot govern the physio-avatar EB system. Therefore, the high CoD of the ADP control strategy early in WO suggests the implementation of a motor control strategy close to virtual trajectory control, resembling the averaged AA ratio trajectory at BL. Toward the later stages of WO, divergence from the motor control strategy of virtual-trajectory-like control is seen, and a return to the BL motor control strategy is identified. The extension movements of Participant B under the fixed condition show a different pattern of return to the BL motor control strategy from that under the free condition, although the Avg of CoD (BL) values under both conditions are similarly low. Therefore, Initial Value (BL) and Avg of CoD (ADP) indicate a deviation from the virtual trajectory control strategy from the early WO period.

The presence or absence of aftereffects from the WO control strategy compared to the ADP control strategy was examined. The continuation or divergence of the ADP motor control strategy during WO may indicate that the ADP control strategy was maintained as an aftereffect during WO. The divergence of the results from the ADP control strategy during the flexion and extension movements of Participant B (Free), where a Time Constant (ADP) was calculated, and the fact that Initial Value (ADP) exceeded Constant Value (ADP) suggest an aftereffect of returning from a virtual-trajectory-control-like control strategy to a non-virtual-trajectory-control-like control strategy. However, for flexion movements, despite the observed divergence from the ADP control strategy, the maintenance of the BL motor control strategy from the early WO period does not definitively indicate aftereffects. Hence, this is considered an example where aftereffects did not occur. Participant A maintained the ADP control strategy during flexion and extension movements under both hand conditions. However, they returned to the BL motor control strategy from the early WO period. Therefore, rather than maintaining the ADP motor control strategy during WO, they controlled the avatar while implementing the BL motor control strategy, leading to a high CoD of the ADP control strategy during WO. Thus, aftereffects did not occur in these four examples. For Participants C and D, the return to the BL motor control strategy from the early WO period and the divergence from the ADP control strategy indicate the absence of aftereffects.

In summary, aftereffects were observed in the results of Participants B (Fixed), C, and E during flexion and extension movements and Participant B (Free) during extension movements, suggesting the effectiveness of the physio-avatar EB intervention. Among the four experiments showing intervention effects, three had SoA scores exceeding 4, and two had SoO scores above 4, with their averages being 4.5 and 3.5, respectively. The degree of SoA may influence the effectiveness of the physio-avatar EB intervention. Prior researchers on SoA and SoO developed methods of artificially creating conditions with SoO but without SoA, or *vice versa*, and found that only SoA is associated with improved motor skills. Therefore, enhancing SoA can be effective in rehabilitation systems (Matsumiya, 2021).

The results of the current study are similar to those of previous studies. With an increased sample size, future research should explore the relationship between NMSS model parameters or experimental conditions and SoA and verify whether the intervention effects are related to SoA and not dependent on SoO. In addition, the results were compared with those of previous studies. No error learning was observed for the rehabilitation device with the EMG and a virtual arm in existing studies (Gieser et al., 2017; Yang et al., 2017; MINDROVE, 2024; Suryanarayanan and Reddy, 1997). Furthermore, the research group cited in the introduction, which had reported that error learning did occur, did not use EMG to generate errors in the muscle space (Lo et al., 2010; Liu et al., 2018; Wei et al., 2005; Abdollahi et al., 2013; Porta et al., 2021). This is because, in these studies, errors such as the finger-tip position error were introduced into the task space. The greatest contribution of the present study is that it confirms that error learning does, in fact, occur in the muscle space when avatars that can be manipulated with EMG signals continuously and in real time are used.

Future challenges include issues with the system and analytical methods. In terms of system-related issues, forearm pronation and supination are important for elbow flexion–extension movements when the shoulder joint is not horizontal. However, in this system, while the avatar is limited to 1-DOF movements within a horizontal plane, the actual body assumes an internal rotation position at the shoulder joint, failing to realize complete horizontal-plane movement. SoA requires a match between the prediction of action outcomes and the feedback of actual results (Watanabe and Nobuyuki, 2016), and the inability to achieve a complete horizontal-plane movement in this work may have reduced SoA values. Future developments should consider the relationship between the shoulder joint posture and movement direction during flexion movements to achieve complete horizontal-plane movement. Regarding issues related to the analytical methods, the discussion centers around the AA ratio trajectory and determining the degree of change compared to before the physio-avatar EB intervention based on the CoD. This article focuses on whether the control strategy returns to the pre-intervention state immediately after the intervention. Results that do not indicate a return to the pre-intervention control strategy are not discussed and should, therefore, be further explored. Additionally, as the CoD may be insufficient for evaluating the trajectory shape, other indicators should be introduced for evaluating trajectories. These issues will be addressed in future work, which will also conduct further verification with healthy individuals and evaluate the clinical efficacy of using the developed system with actual patients.

## 5 Conclusion

In this study, we explored the possibility of changing motor control strategies by applying the physio-avatar EB system to healthy individuals as a case study. This is a preliminary step toward confirming the applicability of the developed system to patients. The intervention, providing an experience of a body different from one’s own, was conducted on seven participants using a time-invariant calculation algorithm to determine the avatar joint angles. The participants’ motor control strategies for identical cyclical movements before the intervention (five sets) and immediately after the intervention (five sets) were compared. Additionally, fixed and free-hand conditions were set during ADP, so 12 experiments were conducted. The data from seven experiments were evaluated after excluding data believed to have been affected by EMG acquisition issues.

The evaluation focused on adaptation to the physio-avatar EB system, the effect of physio-avatar EB intervention, and the impact of SoA and SoO on the intervention effect. Evaluations were conducted separately for extension and flexion movements. To evaluate adaptation to the physio-avatar EB system, the BL and ADP variability indices for the equilibrium point (a control variable of the physio-avatar EB system) and stiffness (a non-control variable) were determined. The degree of increase from BL to ADP was assessed, and findings confirmed such adaptation in all but one experiment. The adaptation indicators of the experiment that failed to confirm adaptation may have been undervalued. In the evaluation of the physio-avatar EB intervention effect, the BL and ADP control strategies for the AA ratio, an indicator of the equilibrium point in human motor control, were determined. The similarity of these control strategies during WO was assessed using the CoD. Exponential approximation of the CoD compared to the control strategies at BL and ADP during WO was performed to determine the time constant, initial value, and convergence value of the exponential approximation. The results then served as indicators for assessing the intervention effect. Changes in motor control strategies due to physio-avatar EB intervention were observed in four experiments, which showed a gradual return to the pre-intervention control strategy. Thus, the physio-avatar EB system affected motor control strategies. Furthermore, the four experiments showing an intervention effect suggest that SoA may influence the presence or absence of such an effect. The employed system specs were considered sufficient when compared with existing studies and required specifications.

## Data Availability

The raw data supporting the conclusions of this article will be made available by the authors, without undue reservation.
